# Abortive vampire bat rabies infections in Peruvian peridomestic livestock

**DOI:** 10.1371/journal.pntd.0008194

**Published:** 2020-06-29

**Authors:** Julio A. Benavides, Andres Velasco-Villa, Lauren C. Godino, Panayampalli Subbian Satheshkumar, Ruby Nino, Elizabeth Rojas-Paniagua, Carlos Shiva, Nestor Falcon, Daniel G. Streicker

**Affiliations:** 1 Departamento de Ecología y Biodiversidad, Facultad de Ciencias de la Vida, Universidad Andrés Bello, Santiago, Chile; 2 Institute of Biodiversity, Animal Health and Comparative Medicine, College of Medical Veterinary and Life Sciences, University of Glasgow, Glasgow, Scotland, United Kingdom; 3 Centro de Investigación para la Sustentabilidad, Facultad de Ciencias de la Vida, Universidad Andrés Bello, Santiago, Chile; 4 Division of High-Consequence Pathogens and Pathology, National Center for Emerging and Zoonotic Infectious Diseases, Centers for Disease Control and Prevention, NE, Atlanta, Georgia, United States of America; 5 Colegio Médico Veterinario de Apurimac, Abancay, Peru; 6 Association for the Conservation and Development of Natural Resources, Lima, Peru; 7 Faculty of Veterinary Medicine and Zootechnics, Universidad Peruana Cayetano Heredia, Lima, Peru; 8 MRC-University of Glasgow Centre for Virus Research, Sir Henry Wellcome Building, Glasgow, Scotland, United Kingdom; International Atomic Energy Agency, AUSTRIA

## Abstract

Rabies virus infections normally cause universally lethal encephalitis across mammals. However, ‘abortive infections’ which are resolved prior to the onset of lethal disease have been described in bats and a variety of non-reservoir species. Here, we surveyed rabies virus neutralizing antibody titers in 332 unvaccinated livestock of 5 species from a vampire bat rabies endemic region of southern Peru where livestock are the main food source for bats. We detected rabies virus neutralizing antibody titers in 11, 5 and 3.6% of cows, goats and sheep respectively and seropositive animals did not die from rabies within two years after sampling. Seroprevalence was correlated with the number of local livestock rabies mortalities reported one year prior but also one year after sample collection. This suggests that serological status of livestock can indicate the past and future levels of rabies risk to non-reservoir hosts. To our knowledge, this is the first report of anti-rabies antibodies among goats and sheep, suggesting widespread abortive infections among livestock in vampire bat rabies endemic areas. Future research should resolve the within-host biology underlying clearance of rabies infections. Cost-effectiveness analyses are also needed to evaluate whether serological monitoring of livestock can be a viable complement to current monitoring of vampire bat rabies risk based on animal mortalities alone.

## Author summary

Rabies virus is the deadliest virus affecting mammals. In Latin America, rabies transmitted by vampire bats is one of the most recognized zoonoses affecting humans and livestock. For unknown reasons, species such as bats and cattle can produce antibodies against rabies that clear viral infection prior to the development of deadly clinical signs. However, the extend of this phenomenon in naturally exposed populations remains unknows. In this study, we detected anti-rabies antibodies among cattle and, for the first time, goats and sheep bitten by vampire bats across as large geographic area endemic of vampire bat rabies in South Peru. We also showed that the presence of antibodies in livestock can indicate the past and future levels of cattle mortality due to rabies in the area. Future research should resolve the within-host biology underlying clearance of rabies infections and evaluate if antibody detection of healthy animals can be used for monitoring vampire bat rabies risk.

## Introduction

Rabies virus (RABV, Family Rhabodoviridae, Genus *Lyssavirus*) is a nearly globally distributed zoonosis that infects all mammals and has the highest case fatality rate of any infectious disease [1]. Natural animal reservoirs include bats and carnivores. Typical rabies infections are transmitted by bite through saliva of an infected individual. The virus disseminates from the bite location to the central nervous system (CNS) via the peripheral nervous system. Neurotropism protects the virus from host immune defenses following the earliest stages of the infection [2,3]. Infections that reach the CNS are almost invariably lethal in both natural reservoirs and accidental hosts (e.g., livestock and humans) [4].

For reasons that remain unresolved, some RABV exposures lead to the production of rabies virus neutralizing antibodies (RVNAs) that clear viral infection prior to the invasion of the CNS and onset of neurological disease [5–7]. These so-called “abortive infections” have been widely documented in apparently healthy wild caught bats and in captive bats following experimental RABV challenge [8–10]. Abortive infections have been hypothesized to occur predominately in bats as a potential consequence of their long evolutionary relationship with RABV [11]. However, the detection of RVNAs in a variety of non-bat species has raised the possibility that abortive infections could be widespread in both reservoir and non-reservoir hosts [11,12]. RVNAs have been reported in apparently healthy and unvaccinated non-human primates, opossums and wild canids [10,13–16] and following experimental infections of laboratory animals [17]. Abortive infections also occur in humans with routine contacts with rabies reservoirs [18–21] and in cattle bitten by vampire bats [22].

Widespread abortive RABV infections open the possibility of using serological studies in non-reservoir species to complement existing surveillance systems to gain better understanding of spatial and temporal patterns of rabies risk. Active serological monitoring would overcome several limitations of existing surveillance systems that rely on passive reports of human and animal mortality or active surveillance of reservoirs. First, serology can overcome the data sparseness and reporting bias often observed in reports of mortality [23]. Second, serological monitoring of non-reservoir hosts might be more practical than serological or virological surveillance of reservoirs, which involves costly capture, sampling and possible euthanasia of elusive wildlife. Furthermore, diagnostics based on pathogen detection (e.g., RT-PCR or antigen detection tests) provide a very low return on effort due to the low incidence of rabies in free-ranging wildlife (typically <1%). Indeed, serological testing is used as a main tool for the surveillance of several other zoonotic diseases in both livestock and wildlife including Brucellosis and Rift Valley fever virus [23–26].

In Latin America, common vampire bats (*Desmodus rotundus*) are the primary source of rabies in humans and domestic animals [27]. The disease, considered among the most important zoonoses for human and animal health in the region, carries a substantial public health and economic cost [28]. Thousands of livestock mortalities are estimated to cost $30 million annually, before considering major investments in culling bats [13,29,30], vaccination of humans and livestock, diagnostics and surveillance programs across Latin America [29,31–33]. Current management actions include preventive vaccination of livestock, post-exposure prophylaxis of humans and control of vampire bat populations using anticoagulant poisons [34]. Estimates of the dynamics of the disease and the identification of risk areas are generally extrapolated from livestock and human deaths reported to national surveillance systems by the public [35,36]. However, reporting efforts vary geographically, creating potential for mis-allocation of resources to areas with high reporting but low incidence [29].

Here, we studied patterns of abortive and lethal rabies infections in peridomestic livestock that are routinely bitten by vampire bats. We focused on an area in southern Peru where all livestock rabies cases are attributed to viral variants which are maintained by common vampire bats [36,37]. This implies that the presence of antibodies in livestock would most likely be explained by viral exposures mediated by the feeding behaviour of infected vampire bats. We focused on three regions which were previously reported to have high rabies incidence and low vaccination rates [29]. We aimed to identify which common livestock species show detectable frequencies of abortive infections and to evaluate whether seropositivity is spatiotemporally correlated with livestock rabies mortality events observed through the passive surveillance system.

## Methods

### Ethics statement

The study was approved by the Ethics Committee of the College of Veterinary Science, University of Glasgow, which covers for animal handling and blood collection (protocol number: 200140112). All participants were read a consent form including study objectives, risks and benefits of participation, confidentiality and that participation was voluntary. Participants received clarification if requested before signing, as well as a leaflet explaining the project, a copy of their written consent and contact information to request study results.

### Study area and sampling design

**Fig 1:**
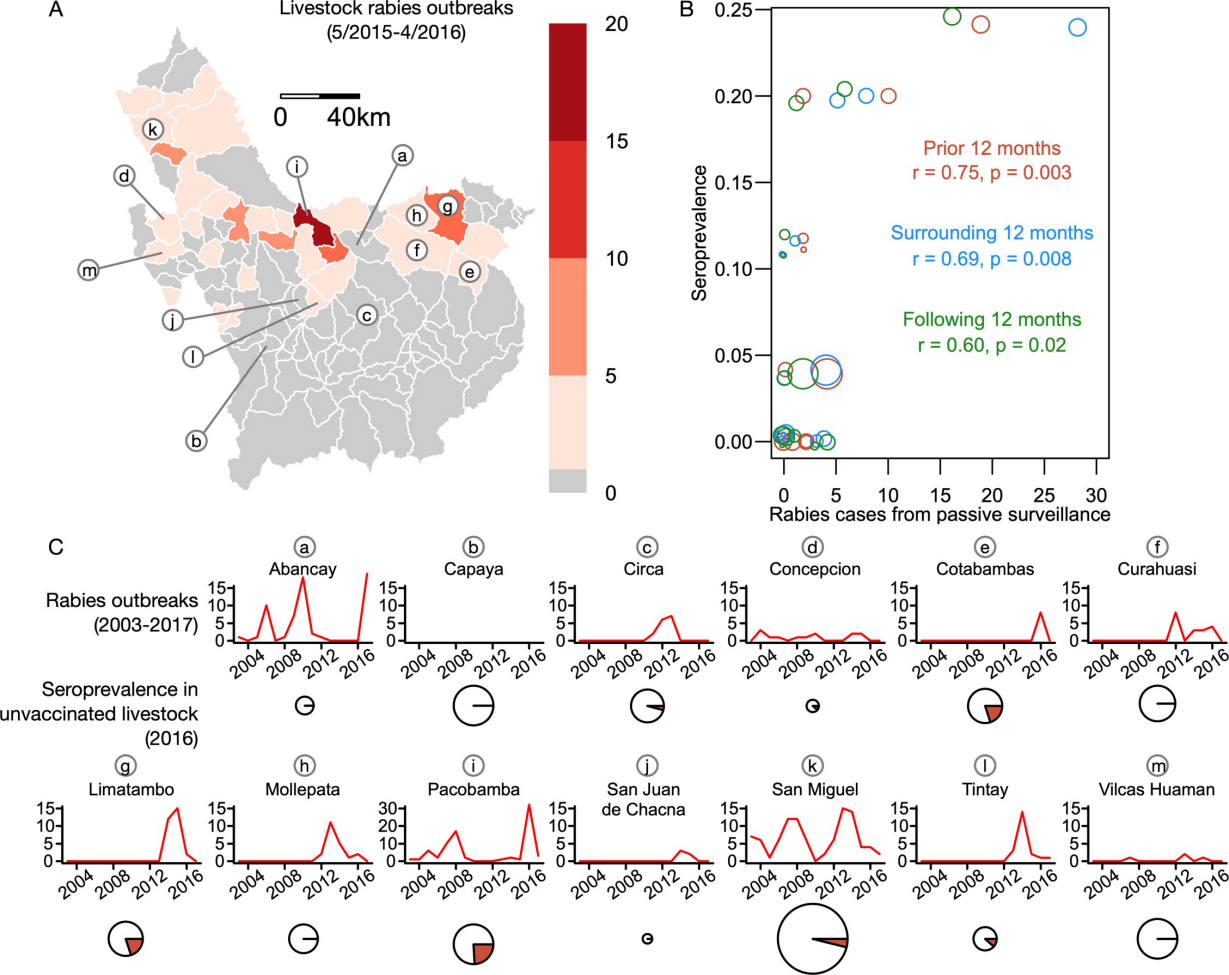
Spatiotemporal correlations between rabies mortality incidence and seroprevalence. (A) Map showing the districts of Peru where the study was carried out, color coded by the number of livestock rabies outbreaks reported to SENASA during 12 months prior to serological sampling. Lower-case letters correspond to districts shown in panel C. (B) Scatterplot showing the relationship between seroprevalence and rabies cases in the 12 months prior to sampling, in the 6 months surrounding sampling, and in the 12 months following sampling at each district. The size of points is scaled to sample size per district (range = 7–51 samples). The correlation coefficient (r) and its associated p-value of a Pearson’s correlation test are shown for each relationship. (C) Time series of rabies incidence in each sampled district from 2003–2017 showing sporadic outbreaks. Pie charts below indicate the proportion of livestock in each district that were seropositive in 2016 (red = positive, white = negative).

Livestock sampling was conducted between May and June 2016 in the regions of Apurimac, Ayacucho and Cusco, Peru, which together account for almost 70% of rabies cases in Peruvian livestock (Fig 1A) [35]. These regions include districts where livestock rabies cases have never been reported (i.e., Cayapa district), districts that experienced recent cases of rabies in the last 2–3 years, and districts where rabies has been endemic during the last decade [35]. These three regions have a livestock population of around 2.4 million sheep, 1.1 million cattle, 290,000 equines (horses and donkeys), 258,000 pigs and 150,000 goats according to the 2012 Agricultural Census of Peru (CENAGRO IV). These species constitute the main food source of vampire bats in this region [38,39]. Vaccination of cattle against rabies shows extensive geographic variation in this region. On average, 58% of farms report vaccinating their animals, but this ranges from 0% of farms in putatively rabies-free districts to 100% of farms in rabies-endemic districts [29]. However, not all animals on vaccinated farms are vaccinated. According to local authorities of the National Agrarian Health Service (SENASA) in Apurimac, species such as sheep and goats are rarely, if ever, vaccinated.

Communities for this study were selected using a stratified random sampling design. We selected 16 districts in the regions of Apurimac, Ayacucho and Cusco (N = 15 rabies infected and N = 1 rabies free; comprising 35 communities) which were accessible by public transport. Within each community, we obtained a list of households that kept livestock from the local community leaders and randomly selected 10 farms asking owners whether they had animals that were never vaccinated against rabies. We collected serum samples from livestock from 1 to 9 farms within each community in order to sample up to 30 unvaccinated animals per community. Given the high burden associated with rabies in cattle [29], priority was given to sample cattle over other livestock species. Sampled farms contained an average of 10.6 cows per farm and a lower number of other animals.

### Questionnaires

Each farmer answered a short oral questionnaire related to each animal sampled including its vaccination history, age, bite status, past and current disease symptoms and treatment history. Animals were visually inspected for the presence of past and fresh vampire bat bites by the technicians performing the questionnaires. Farmers name each animal and can individually recognize them. Each animal’s characteristics were recorded during the questionnaire and were used for later identification of animals during follow up investigations of potentially seropositive individuals.

### Serum sampling

For blood collection, animals were restrained by at least three technicians using ropes. Blood samples were taken from the jugular or the tail, depending on the most accessible location for each animal. Samples were collected in 5mL glass tubes with no additive and kept at 4°C until centrifugation (within 5 hours). Up to 1.5mL of serum was transferred to 2mL cryotubes. Cryotubes were transported on dry ice to the Universidad Peruana Cayetano Heredia in Lima, where they were heat-inactivated at 62°C for 2 hrs to eliminate risks of foot and mouth disease virus (per USDA-APHIS import regulations). Tubes were then stored at -80°C prior to shipment to the United States Centers for Disease Control and Prevention (APHIS import permit number 132155).

### Laboratory analyses

Sera were tested for RVNA using the rapid fluorescent focus inhibition test (RFFIT) [40]. All samples were screened for the presence of RABV using a 1:5 dilution of serum [22]. Samples with 100% neutralization of the virus at 1:5 were considered seropositive. Positive sera were tested in duplicate to determine end-point titers. Raw titers were converted to international units (IU) by comparing the sample against a titer of a standard rabies immune globulin (SRIG) at 2 IU/mL using the following formula: $Titer\;in\;IU/mL=\frac{sample\;titer}{reference\;serum\;titer}\times value\;of\;the\;reference\;serum\;in\;IU/mL$. Laboratory results are summarized in S1 Table of Supplementary Material.

### Statistical analyses

Prevalence of seropositive animals was reported for each livestock species, and confidence intervals were calculated using the *binom*.*confint* function (Agresti-Coull method) in the binom package in R 3.2.1 [41]. Statistical models evaluated the association between serological status and several variables including livestock traits (species [using cattle as the reference factor], sex, and age), bite status (presence or absence of past or fresh bites) and three variables related to the occurrence of rabies in each district: the number of livestock cases reported in that district one year before sampling (LR1), the number of cases reported 6 months before and 6 months after sampling (LR2), and the number of cases one year following sampling (LR3). LR1, LR2 and LR3 data were calculated from the monthly number of laboratory-confirmed livestock rabies cases reported by SENASA. Although reports of mortality at the community level would be expected to most accurately reflect the risk of VBR exposures to livestock in a community [29], community level data were too sparse for our analysis due to widespread under-reporting of rabies mortality and/or the low incidence of rabies in some communities. We therefore conducted these analyses using rabies reports aggregated at the district level. Age was evaluated as a quantitative value (number of years), as a binary variable (greater or less than one year), or as a categorical variable with 4 age classes (<1 year, 1–5 years, 5–10 years or > 10 years). Each version of the age variable was tested in a separate model keeping all other variables the same. For ease of presentation, results for other (non-age) variables are shown using the quantitative value of number of years, but results were robust to the alternative age definitions.

The binary nature of our response variable (i.e., positive or not) and the possibility of community-level differences, required using generalized linear mixed models with binomial errors (i.e., logistic regression). Analyses used a generalized quasi-likelihood linear mixed models (glmmPQL) including the identity of the community as a random effect. All models were built using the glmmPQL function of the MASS package in R. Quasi-likelihood models were preferred to other maximum-likelihood methods (i.e., lmer package) since the low number of positive samples and values of some categories (e.g., N = 5 pigs) challenged the convergence of those models. The statistical significance of each variable was assessed using the Wald t-test. Since variables LR1, LR2 and LR3 were correlated (Pearson’s correlation test for LR1 and LR3, Rho = 0.92, p < 0.001, Pearson’s correlation test for LR2 and LR3, Rho = 0.97, p < 0.001), we only focused on results from LR1, which did not differ qualitatively from models using LR2 or LR3.

## Results

A total of 332 animals were sampled and serology was successfully performed on serum samples from 305 animals from 13 districts (92% of samples). The remaining samples contained high levels of cytotoxicity, precluding credible assessment of RVNAs. The number of animals sampled per species and their respective laboratory results are provided in Table 1. Fifty-seven percent of samples were from cattle (Table 1). Twenty-three animals out of 305 were seropositive with titers >0.10 UL/mL, for an overall seroprevalence of 7.5% (95% CI: 5–11), 11% (95% CI: 7–17) for cattle, 5% (95% CI: 1–15) for goats and 3.6% (95% CI: 0–20) for sheep. Neither equines (N = 38) nor pigs (N = 5) were seropositive. Titers from positive animals ranged from 0.12 to 70 IU/mL (Table 1). Three cows from three different districts had titers >40 IU/mL. Five farms had more than one seropositive animal, including a farm where both a sheep and a goat were seropositive. Twelve of the 35 communities sampled (34%) had at least one seropositive animal. All animals were assessed as healthy during sampling and questionnaires confirmed the lack of recent instances of illness. Follow up activities carried out 2 years after sampling confirmed that none of the seropositive animals had died from rabies.

**Table 1:** Characteristics of seropositive animals.

Animal	Number of Seropositives over total number of samples	Age	Sex	Fresh bite	Previously bitten	District	End-point Titer	End-Point IU/mL
**Cattle**	**19/173**	** **						
cow_1		5 years	F	yes	yes	Cotabambas	01:45	0.5
cow_2		3 years	M	yes	yes	Cotabambas	01:50	0.55
cow_3		5 month	F	yes	no	Cotabambas	01:56	0.62
cow_4		4 month	F	yes	no	Tintay	1:280	3.1
cow_5		3 month	M	no	yes	Pacobamba	1:21	0.23
cow_6		2 years	F	yes	yes	Pacobamba	1:11	0.12
cow_7		2 years	F	yes	yes	Pacobamba	1:56	0.62
cow_8		1 years	M	yes	yes	Pacobamba	1:210	2.3
cow_9		6 years	F	yes	yes	Pacobamba	1:125	1.4
cow_10		4 month	F	yes	no	Pacobamba	1:6300	70
cow_11		2 month	M	no	yes	Pacobamba	01:22	0.21
cow_12		3 month	F	no	yes	Limatambo	1:4200	46.7
cow_13		3 month	F	no	yes	Limatambo	1:280	3.1
cow_14		2 month	M	no	yes	Limatambo	1:280	3.1
cow_15		5 month	M	no	yes	Limatambo	1:56	0.62
cow_16		3.5 years	F	no	yes	Limatambo	1:11	0.12
cow_17		1.5 years	M	yes	no	Concepcion	1:5700	63.3
cow_18		9 years	F	yes	yes	San Miguel	01:22	0.21
cow_19		2 years	F	yes	no	Circa	1:112	1.1
**Goats**	**3/60**	** **						
goat_1		6 month	F	no	yes	Cotabambas	01:11	0.12
goat_2		2 years	F	no	no	Tintay	01:11	0.12
goat_3		2 years	M	yes	yes	San Miguel	1:280	3.1
**Sheep**	**1/28**	** **						
sheep_1		3.5 years	F	no	yes	Cotabambas	01:56	0.62
**Horses**	**0/38**	** **						
**Donkeys**	**0/1**	** **						
**Pigs**	**0/5**	** **						

Fresh vampire bat bites were observed in 56% of all animals, while 77% had evidence of older bites. Overall, 92% of sampled animals presented evidence of bite exposures on the neck, back or extremities. The frequency of fresh bites was similar in females (55%) and males (58%), as well as in animals older (56%) and younger (58%) than one year. Because nearly all animals were bitten, we lacked statistical power to detect potential effects of bat bites (even only fresh bites) on seropositivity (Odds Ratio [OR] = 0.72, p = 0.69). None of the age variables were correlated with serological status. Likewise, 63% of all animals where females, but animal sex did not influence serological status (OR = 0.76, p = 0.46). Regarding differences between host species, only sheep were less likely to be seropositive than cattle (OR = 0.18, p = 0.04).

Data on reported livestock rabies incidence revealed distinct epidemiological histories among the 13 districts included in this study. Districts ranged from long-term rabies-free areas but experiencing vampire bat bites on livestock (i.e., Capaya), to areas that experienced recent cases of rabies in livestock (e.g., Circa, Limatambo, Tintay) and historically endemic areas that experienced multiple outbreaks interspersed by apparent viral extinctions (e.g., Abancay, Pacobamba, San Miguel; Fig 1C). Consequently, at the time of sampling, sites presented variation in the contemporaneous occurrence RABV and in the time since viral circulation had last been detected (Fig 1A). Only one community with seropositive animals was in a district (Circa) without a confirmed case of rabies reported to the national surveillance system in the 12 months prior our sample collection. However, outbreaks had occurred in that district in earlier years (Fig 1C). No seropositive animals (0 out of 29) were found in the Cayapa district where farmers had never reported VBR rabies in livestock. The number of cases reported one year prior to sampling (LR1) increased the likelihood of being seropositive (OR = 1.18, p < 0.01, Fig 1B). The number of cases reported 6 months prior or after sampling (LR2) (OR = 1.11, p = 0.01) and the number of cases reported one year following sampling (OR = 1.16, p = 0.03) also increased the likelihood of being seropositive.

## Discussion

We found that unvaccinated cattle, goats and sheep that are regularly bitten by vampire bats produce detectable levels of RVNAs and remained healthy for at least two years after sampling. RVNAs were previously reported in 12% of cattle that were bitten by vampire bats in two farms of Guatemala [22]. Our results confirm similar levels of seroprevalence (11% in cattle) in a larger geographic area in Peru. In addition, we report RVNAs in sheep and goats, which we interpret as new evidence of abortive infections in these species. The correlation of abortive infections with reported cases of livestock mortality highlights the possibility of using livestock serology to approximate levels of rabies circulation in bats and suggest that rabies mortality reports alone do not fully characterize rates of viral exposures from bats to livestock.

The seropositive animals we detected are unlikely to reflect ‘false positives’ in the RFFIT test. Cross neutralization of RABV with antibodies elicited by other (non-Lyssavirus) infectious agents or non-antibody mediated immune effectors cross neutralizing rabies infection by precluding viral entry to cells has not been reported or speculated previously and no Lyssaviruses other than RABV circulate in the Americas. Although we did not have true seronegative controls (i.e., sera from animals known never to have had rabies exposures) the twenty-nine seronegative animals from the Cayapa district, where rabies has never been reported before, could be considered as a rough approximation of titers expected in such a control population. We also lacked seropositive control animals for abortive infections (i.e., livestock known to be exposed to confirmed rabid bats) and it is unclear how such controls could be obtained in field conditions. However, complete neutralization at the 1:5 dilution is unlikely to be explained by any mechanism other than rabies neutralizing activity and the presence of higher titers provides greater confidence of RVNA presence.

The presence of RVNAs in healthy, unvaccinated livestock in our study is most likely explained as abortive infections following bites from rabies-infected common vampire bats. One alternative explanation is that seropositive animals had an unreported history of vaccination [22]. Unfortunately, formal records of vaccination histories are not routinely kept in the communities we sampled and absence of vaccination would not be documented. Longitudinal studies following unvaccinated sentinel animals for several years after birth would increase certainty regarding the absence of vaccination, but the remote location of most farms and financial constraints made long term monitoring impossible. Nevertheless, our observation of seropositivity not only in cattle, but also sheep and goats (which are rarely if ever vaccinated in our study area) provides strong evidence that unreported vaccination is unlikely to explain our results. Another possible explanation for the origins of RVNAs in unvaccinated livestock is exposures to RABV variants which are not maintained by vampire bats. Dog rabies has been eliminated from this region, but other RABVs may circulate in insectivorous bats as observed elsewhere in South America [42,43]. It is also conceivable that currently undiscovered variants circulate in wild carnivores. However, the absence of non-vampire bat-associated viruses in hundreds of rabies positive brains that have been typed from this region over more than 15 years (1997–2012) makes these hypotheses unlikely [36,37]. Our results therefore strongly favor exposures to rabies from vampire bat bites as the primary source of abortive infections.

The survival mechanisms and state of protective immunity of individuals with abortive RABV infections remain unknown. Current hypotheses to explain abortive infections include factors related to exposure (dose or anatomical location of bite), lower virulence of RABV variants associated with bats relative to carnivores due to enhanced replication outside of the CNS, as well as higher resistance of bats due to their long evolutionary relationships with Lyssaviruses [11,12]. Our study, and several others describing naturally-occurring RVNAs in non-reservoir species, suggests that abortive infections are a universal phenomenon regardless of species evolutionary relationships with RABV. Abortive infections may therefore arise through particular virological (viral variant) or epidemiological features related to exposure, rather than being explained by host-specific differences in innate viral immunity or in the capacity of the immune system of bats to resolve viral infections. Another outstanding question is whether naturally acquired RVNAs are protective against future exposures. Future longitudinal studies could evaluate the duration of RVNAs following natural exposures while experimental inoculations with RABV could evaluate whether abortive infections induce protective immunity. Finally, the individual-level traits that predict why some livestock succumb to lethal infections while others survive without clinical signs remain unexplored. Future studies should explore whether individual traits related to immunological responses such as T-cell levels could be related to rabies survival without development of clinical signs.

Our statistical models revealed higher seroprevalence in cattle than sheep. Interestingly, this discrepancy mirrors the prey preference of vampire bats in Peru according to blood meal analysis [38]. Moreover, epidemiological data indicate that cattle represent around 90% of reported rabies cases in Peruvian livestock [29]. Therefore, lower seroprevalence in sheep is most parsimoniously explained by lower incidence of bat bites arising from vampire bat feeding preference rather than inter-specific differences in susceptibility. The lack of correlation of seroprevalence with other factors, including the age and sex of animals, is consistent with our finding that vampire bats fed on livestock across all demographic groups and further suggests an absence of dramatic differences in the probability of lethal or abortive infection among age/sex groups.

The strongest predictor of RVNAs in livestock was the number of rabies deaths reported one year prior to sampling (Fig 1B). This suggests that seroprevalence in livestock reflects the recent levels of RABV risk in livestock, which is expected to be directly linked to the circulation of RABV in vampire bat populations. The number of rabies deaths reported one year following sampling was also correlated with RVNAs in livestock, suggesting that surveillance of RVNAs could provide additional information to predict the future risk of rabies exposures to domestic animals and humans at the district level. Seroprevalence data might therefore complement existing surveillance systems that rely on animal mortality and bite incidence to identify high risk localities for control, as implemented for other zoonotic diseases [24,26]. Such data could be particularly useful to refine quantifications of RABV risk in areas where reporting rates to passive surveillance systems are low, enabling identification of risk even in areas without official reports of cases. The duration of RVNAs following exposure to rabies, which is yet to be determined for these livestock species, may identify the specific time window associated with RABV exposures assessed from serological data. Despite its potential utility, the viability of implementing sero-surveillance in low-income settings will have to overcome several current challenges including the current high cost of RFFIT, limited laboratory capacity to perform the test in regional and national laboratories and lack of training of personnel on sampling collection, transport and storage. These challenges might restrict the implementation of this technique only to epidemiological scenarios where the cost of surveillance would outweigh the consequences of rabies circulation, e.g., surveillance of ´rabies-free´ districts with neighbouring districts reporting cases. Alternative serological techniques such as high-throughput binding assays (e.g., ELISA) could alleviate some of these challenges, but are limited by their lower sensitivity, inability to determine whether antibodies are neutralizing, the high cost of commercial kits, and the need to validate the accuracy of kits across sera from different host species.

Our findings also underscore the potential for serological data to facilitate new lines of rabies research. First, future studies could use serological data to identify risk factors such as animal husbandry conditions or land use practices that increase the risk of RABV exposures. Serological data from livestock might inform also parameters required for mathematical models of rabies transmission between vampire bats and non-reservoir hosts [44–46]. For example, serological data from vampire bats allowed inference of the mechanisms underlying rabies circulation in bat populations, including virus dispersion between colonies and a high frequency of abortive infections [47]. Adding serological data from livestock could be used analogously to inform the spatial and temporal patterns of rabies incidence in bats in areas where capture and sampling of bats is difficult, undesirable or cost-prohibitive. These data could also inform rates of transmission between species, a key gap in most models of emerging viruses [48]. Finally, serological monitoring of livestock represents a new option for monitoring the effectiveness of measures aiming to reduce rabies circulation among bats (e.g., culling or vaccinating bats) [13,49,50] which overcomes the sparse data limitation that arises from exclusively relying on reported mortalities and the logistical challenges of sampling large numbers of wild bats.

In summary, this study demonstrated RVNAs in multiple livestock species which are best explained by abortive infections following bites from infectious vampire bats. Patterns of seroprevalence largely mirrored vampire bat feeding preferences, suggesting a similar probability of abortive infection following rabies exposures across species, age groups and sexes. Understanding the basis for abortive infections remains an outstanding gap in rabies biology which might eventually inform novel therapies for rabies treatment. At the epidemiological level, integrating serological data from non-reservoirs into rabies surveillance may refine our understanding of rabies dynamics and local risk, ultimately enhancing options for preventative action.

## Supporting information

S1 Table Serology results for each animal tested. (XLSX)
